# Improving access to health care amongst vulnerable populations: a qualitative study of village malaria workers in Kampot, Cambodia

**DOI:** 10.1186/s12913-017-2282-4

**Published:** 2017-05-08

**Authors:** Marco Liverani, Chea Nguon, Ra Sok, Daro Kim, Panharith Nou, Sokhan Nguon, Shunmay Yeung

**Affiliations:** 1 Department of Global Health and Development, London School of Hygiene and Tropical Medicine, London, UK; 2 National Center for Parasitology, Entomology & Malaria Control, Ministry of Health, Phnom Penh, Cambodia; 3 Partners for Development, Phnom Penh, Cambodia; 4 Department of Clinical Research, London School of Hygiene and Tropical Medicine, London, UK

**Keywords:** Cambodia, Community health workers, Malaria, Health equity, Health seeking behaviour, Access to health services, Mobile populations, Forest workers

## Abstract

**Background:**

There is growing interest in the expansion of community health workers programmes in low- and middle-income countries as a cost-effective approach to address shortages of health professionals. However, our understanding of the reception of large-scale programmes and how to improve them remains limited, with knowledge gaps about factors that may promote or discourage equitable access to services. This paper examines the case of the Village Malaria Workers (VMW) programme in Cambodia, an extensive community-based intervention for the management of malaria cases in remote rural areas.

**Method:**

Fieldwork was conducted in Kampot province, in six case villages characterised by different programme configuration, population size, and distance to the nearest public health facility. In these locations, in-depth interviews (*n* = 71) with VMWs, village authorities, and residents were conducted to identify facilitators and challenges to service utilisation. Data analysis was informed by a conceptual framework based on five domains of access to services: awareness, accessibility, accommodation, availability, and acceptability.

**Results:**

Factors that influenced the utilisation of VMW services in our research sites include: the nature of dissemination activities and their ability to reach different population groups; the village topography and the changing road infrastructure; the involvement of VMWs in other community roles and activities; perceptions about the type of disease after the onset of symptoms; the need for comprehensive diagnosis and care; perceptions about the status of VMWs as medical providers; length of VMW appointment.

**Conclusions:**

This study highlights the complexity and diversity of contextual factors that may influence the uptake of a community health programme. As in other countries, continued use of lay health workers in Cambodia to deliver diagnostic and curative services has the potential for great health and economic impact. However, further consideration should be given to the problem of access in different categories of residents and different contexts of implementation. In addition, a comprehensive mapping of changes in disease epidemiology, road infrastructure and the geography of access to services is crucial to inform policy development in this area.

**Electronic supplementary material:**

The online version of this article (doi:10.1186/s12913-017-2282-4) contains supplementary material, which is available to authorized users.

## Background

Despite sustained efforts to strengthen the health system and significant progress, Cambodia still suffers from critical shortages of health professionals and inequalities in the distribution of health services [1, 2]. While the number of doctors and clinics has increased in cities and larger towns, gaps remain in the availability and quality of health services in rural areas [2, 3]. This problem is more acute in remote communities, where the incidence of infectious diseases such as malaria is higher, where access to health facilities may be limited by environmental barriers and lack of adequate road infrastructure, and where poor communities bear the greatest economic burden of ill health.

The involvement of lay members of the community to provide basic health services has been one approach to address this problem. Volunteers have been trained to support a range of interventions in their communities, including health promotion and education, child nutrition, maternal and reproductive health, tuberculosis, mental health, infectious disease surveillance, and malaria prevention and control [4–9]. In addition, village health support groups (VHSG) have been established to represent the needs and concerns of village residents at committee meetings for the planning, use, and management of local health facilities.

Based on positive evaluations of early programmes, the Cambodian government has emphasised the need for sustained and expanded participation of the community in health care. The Health Strategic Plan (2008–2015) of the Ministry of Health aimed to “strengthen integrated outreach and community-based models of care as an intermediate strategy (…) especially in communities with limited access to health facilities” [10]. Yet, policy and planning in this area have tended to proceed without clear indications on the way in which the scaling up of interventions should be implemented. For example, it is unclear whether health volunteers should mainly serve as a link between the community and the health system (e.g., through surveillance, case referral, health promotion) or they should be involved increasingly in care and treatment delivery, thus taking on a role and tasks which are conventionally performed by health professionals. It is also uncertain whether future programmes should be driven by a vertical approach, focused on specific diseases or health concerns, or health workers should provide a more comprehensive range of services. Most important, knowledge gaps remain about factors that may facilitate access to services. Although a number of surveys and evaluations have been conducted in recent years [4, 7, 11–14], these studies have not been informed by qualitative, in-depth, analyses of the reception of programmes in user communities.

The present paper aims to examine and reflect on these issues by presenting and discussing findings from an exploratory qualitative study of the village malaria workers (VMW) programme, one of the most extensive community-based interventions in Cambodia. This programme was introduced in 2001 by the National Malaria Centre (CNM) in collaboration with the European Commission, as part of a community based trial of insecticide-treated bednets in Rattanakiri province, North East Cambodia. Community volunteers were selected in each study village on the basis of literacy, and trained to diagnose suspected malaria cases by using a rapid diagnostic test (RDT), administer artemisinin-based combination therapy (ACT), and refer severe patients to the nearest public health facility [15]. Following this experience and another pilot project in Koh Kong province, the village malaria worker approach was approved by the Ministry of Health as an emergency intervention for malaria control in remote communities, and rolled-out in 2004 with the financial support of the Global Fund to cover 300 remote and highly endemic villages. In these villages, one “mobile malaria worker” (MMW) was later established in addition to the standard VMW to encourage active detection of malaria cases around the village and in the surrounding areas near the forest fringes where the risk of malaria is higher. All VMWs were expected to contribute to other malaria control activities such as health education and distribution of bed-nets.

Over the years, the VMW programme has been expanded incrementally, currently covering 1602 villages in 17 provinces and a population of about 1.2 million. Unlike the original plan, the new villages have been selected by the distance (>5km) from the nearest health centre, not according to malaria risk. Candidates are shortlisted by the village chief in consultation with the local health centre, and appointed by village election. After appointment, VMWs receive a two-day training at local facilities of the Ministry of Health, with refresher sessions in the following years. Training is also given to one VMW assistant, who is usually a relative living in the same house (e.g., spouse, sibling, child) and can deliver diagnosis and treatment when the principal VMW is not available. Further, VMWs in 400 villages, who had started providing malaria control services by 2008, were also trained to provide treatment for acute respiratory infections (ARIs) and diarrhoea with antibiotics or oral rehydration salts (ORS) and zinc respectively for children under 5 years. All VMWs attend monthly meetings held at the health centre to which are they are linked, where they receive replenishment and supervision.

Our study explored factors that may promote or discourage access to VMW services in poor communities. The problem of access to care has long been the focus of theoretical debate in public health studies, and different concepts and definitions have been developed to support empirical work and analysis. In an early contribution, Penschansky and Thomas [16] defined access as “a concept representing the degree of ‘fit’ between the clients and the system”. Further, they identified five interrelated dimensions of access that could be used to inform systematic research and data analysis: *availability* indicates the adequacy of the supply of health services and products; *accessibility* is the relationship between the location of providers and users, including travelling time and cost; *accommodation* refers to the organisation and management of health facilities, and their capacity to meet users’ need in terms of hours of operation and waiting times; *affordability* indicates the cost of health care and the ability of users to pay that cost; *acceptability* refers to the range of cultural and social factors that may influence the relationship between users and providers, such as age, gender, ethnicity or religious issues. This model has provided methodological foundations to many studies in health services research to date. Concurrently, novel concepts and insights have been developed to account for changes in the wider context of global health policy, such as the introduction of user fees in developing countries and resulting imbalances in health service utilisation. In particular, increasing emphasis has been placed on the notion of *equitable access*, understood as the “conditions whereby those in equal needs have equal *opportunities* to access health care” [17]. In addition, the literature has explored other key factors that may influence access to services amongst disadvantaged groups, such as lack of entitlement [18], constraints related to livelihood insecurity [19], and the acceptability of interventions where conflicts may exist between local and biomedical understandings of disease and treatment [20, 21].

Drawing on this body of work, we considered five dimensions of access as relevant to our study, both in terms of type of intervention (i.e., free of charge, community-based, diagnosis and treatment) and context (i.e., low-income and remote communities): awareness, accessibility, availability, accommodation, and acceptability. Following Penschansky and Thomas [16], we recognise these dimensions are closely related yet sufficiently distinct to provide a useful framework for systematic research and data analysis. In the following sections, we provide details of the methodological approach for this study and the main research findings. We then discuss tensions, limitations and opportunities that should be considered in the scaling-up of interventions, and the implications of our research for global governance in this increasingly important area of health and development policy.

## Methods

Our research for this paper is part of a larger project on the impact of the VMW programme on appropriate treatment for malaria and childhood illnesses amongst the most vulnerable populations in Cambodia, conducted in Kampot province in 2013. In this context, we carried out an exploratory qualitative study to gain a better understanding of care seeking behaviour in the communities and factors that may promote or discourage the utilisation of VMW services.

### Study setting

The total population in Kampot at the time of the study was circa 530,416 inhabitants or around 190,000 households across 437 villages. While socio-economic status and educational level is above the national average, 22.5% of women and 18.3% of men “cannot read at all” and 15.6% of households are below the poverty line [22]. As in the rest of Cambodia, malaria transmission has decreased in recent years [23]. However, risk of infection remains high in forested areas, particularly amongst mobile population groups who are involved in forest-related activities (e.g., logging, clearing) and sleep in temporary forest shelters such as tents or hammocks between trees [24, 25]. Child diseases are important health concerns in Kampot although the incidence of ARIs and diarrhoea in children under 5 is lower than in other provinces [22].

Of the 437 villages in Kampot, 63 villages had VMWs, of which 27 were part of the expanded programme where VMWs could also treat child diseases, and are referred to hereafter as expanded role VMWs or “eVMWs”. In addition, 12 villages were served with MMWs. For the present study, six case villages were purposively selected using the following criteria: (1) higher annual malaria incidence (mean = 19.7), as indicated in the VMW case reports; (2) relatively high poverty level (mean = 22.9%), as measured in the Cambodia’s Database of Poor Households (2011); (3) variable average travel time to and from the nearest public health centre (range = 22.15′–94′); (4) variable population size (mean = 1439; SD = 474). Further, we selected three villages with VMWs providing only malaria diagnosis and treatment and three villages with access to expanded role VMWs.

### Study sample

In each village we aimed to approach and conduct in-depth interviews with different categories of informants to collect multiple voices and experiences on the research topic. Participants included (1) village authorities, (2) VMWs, and (3) potential users of VMW services in the village population. Adult males who worked in forested areas and had malaria in the year preceding the interview were the key target group of potential users in our study. As noted earlier, this is a high-risk category for malaria infection in Cambodia, identified as “a matter of priority throughout the country” in the strategic plan of the National Malaria Center [23]; further, this population group (hereafter “forest goers”) is particularly vulnerable, as poverty is one of the main incentives to seek resources in the deep forest where working conditions are hard and higher risk of malaria exist [24]. Additionally, in eVMW villages, we interviewed mothers of children under the age of 5 (hereafter “caregivers”) who had recently experienced febrile illness, as these were potential users of expanded services. To minimise selection bias, multiple strategies and entry points were used to identify potential participants, including consultations with community leaders, snowball sampling, and by questioning villagers if they knew forest goers and caregivers who would fit the selection criteria.

### Interview approach and data analysis

Our data collection approach was tailored to different categories of informants. Interviews with village authorities aimed to gain an overview of the socio-economic profile of the population, health-seeking behaviour, and their views on the VMW programme. Interviews with VMWs focused on various aspects of their work (i.e., background, dissemination activities, satisfaction, utilisation), with particular attention to perceptions about their role and acceptance in the community. In designing interview guidelines for forest goers and caregivers, we followed a standard method in studies of health seeking behaviour, aimed at reconstructing step-by-step individual pathways of care [26]. Questions about perceptions, beliefs, and motivations were asked at different stages of the interview to elicit responses about the range of factors that may have influenced individual choices (see Additional files 1 and 2). In order to minimise the potential for biases, participants were explicitly questioned about their knowledge and experience of malaria workers only at the end of the interview, unless the respondent raised the subject earlier in the interview. Interviews were piloted at the early stages of research to refine questions in the guidelines. At the end of each interview, a brief structured questionnaire was conducted to collect basic demographic information.

All interviews were conducted face-to-face in Cambodian, recorded, and subsequently transcribed and translated into English. Participants were interviewed at home by a team of four research assistants, all native Cambodian speakers and graduates in the social sciences. The duration of interviews ranged from 30 min to 2 h approximately, with an average duration of about 1 h. Interviews were conducted until a good understanding of care-seeking behaviour and the role and reception of VMWs in the six villages was achieved. Transcripts were coded into themes in an iterative process [27], and structured within the five domains of the access framework. Data were analysed using QSR nVivo 10 software. Observational field notes were taken at each fieldwork site, and used to inform the analysis.

## Results

Overall, we interviewed 10 VMWs, 10 village authorities, 35 forest goers and 16 caregivers (Table 1). None of the individuals we approached refused to participate in the study or dropped out during the interview. A summary of demographic characteristics of participants is provided in Table 2.

**Table 1:** Study sample

Village 1	Village Chief, Deputy Village Chief, VMW, MMW, 7 forest goers
Village 2	Village Chief, Deputy Village Chief, VMW, MMW, 8 forest goers
Village 3	Village Chief, Deputy Village Chief, VMW, 5 forest goers
Village 4	Village Chief, Member of the Village Committee, eVMW, MMW, 6 forest goers, 5 caregivers
Village 5	Deputy Village Chief, eVMW, MMW, 4 forest goers, 6 caregivers
Village 6	Deputy Village Chief, eVMW, 5 forest goers, 5 caregivers

**Table 2:** Characteristics of interviewed participants

	Forest goers (*n* = 35)				Caregivers (*n* = 16)			
	Means	Range	SD	n (%)	Means	Range	SD	n (%)
Age (years)	35.4	15–59	9.8		26.7	21–24	3.9	
Residency at village (years)	17.1	2–43	12.6		7.4	1–13	3.1	
Education								
Some primary				19 (54.3%)				6 (37.5%)
Completed primary				3 (8.6%)				2 (12.5%)
Some secondary				7 (20%)				5 (31.3%)
Never attended school				5 (14.3%)				3 (18.8%)
Own a motorcycle				16 (45.7)				7 (43.8%)
Own a bicycle				24 (68.6%)				5 (31.3%)
Own a mobile phone				29 (82.9%)				11 (68.8%)

### Overview of care-seeking behaviour

Pathways to care in both categories of participants varied, reflecting different needs, preferences, and the diversity of health care supply in the study locations. After the recognition of symptoms, the first step often involved self-medication with drugs purchased at local outlets or traditional remedies such as herbal infusions, massage, or “coining”, a form of skin scraping therapy widely practiced in Southeast Asia [28]. When symptoms persisted, medical care was sought with providers in the public sector (i.e., VMWs, local health centres, and referral hospitals), the private sector (i.e., private clinics, pharmacies, drug shops), or both. Participants who developed malaria symptoms in the forest typically self-medicated with drugs purchased locally; if not recovered, many would go back home to have support from their families and access health care in or around their villages. Factors that were reported to influence care-seeking behaviour included reputation and attitude of the provider, trust, perceptions about the type and severity of illness, proximity, opening hours, and financial considerations. Some participants accessed different points of medical care during the same febrile episode either because the first provider was not available or the patient was not recovered. The private sector was a popular care option for both caregivers and forest goers, with the majority of participants (*n* = 39/51) who reportedly visited one or more private providers during the latest episode of febrile illness. Some respondents explained this preference by noting that private clinics would treat patients quickly, while long waiting time and shorter opening hours were cited as barriers to the utilisation of public health centres. Three village authorities pointed to the importance of wealth in influencing the choice of provider. For example, one village chief said that “rich villagers go to private clinics, the poor go to the VMW or the health centre”, where they could benefit from user fees exemption. However, many participants resorted to borrowing money or selling essential household assets (such as rice or livestock) to pay the costs of private care, suggesting that socio-economic status was not the only predictor of care-seeking behaviour in the study sample.

### Utilisation and reception of VMWs

Most VMWs noted that malaria cases had reduced in their villages, but malaria transmission in the forest was still a concern. In all villages, VMWs reported that the great majority of adult patients who had used their services were young men involved in forest work. Indeed, the majority of forest goers (*n* = 20/35) in our sample had visited the local VMW in the past, and some (*n* = 9/35) had done so in their latest episode of febrile illness. In “eVMW” villages, however, only a minority of caregivers (*n* = 3/16) had used VMW services for their children. In the following sections, we present the range of factors and perceptions that may have promoted or discouraged access to VMWs. Findings are structured around the five domains of access to services, and illustrated with quotations from the interviews. Where appropriate, forest goers and caregivers will be referred to collectively as “participants”.

### Awareness

Most VMWs (*n* = 7) and some authorities (*n* = 3) said that village meetings were an important activity for the dissemination of information about community health services. Meetings took place at the village chief house or in other key social venues such as the local pagoda, the Buddhist temple. VMWs were often invited to attend such events and promote their work. One VMW explained:

*I always introduce myself and my role to people when there is a meeting in the community. [I say] I am the one who does finger prick and treat malaria for free* (V1_VMW)*.*

Four VMWs and two caregivers noted that word-of-mouth communication between kinfolks and neighbours was another way in which information was spread in the communities; as one VMW said, “people come to me as they know each other and talk; so they know my services are free of charge” (V3_VMW). Others stressed they were well-established members of the community, and therefore could promote their work through social networks or other social roles. One expanded role VMW, for example, explained he could do effective promotion because he was an *achar*, a lay ritual specialist who is responsible for the organisation of weddings and other ceremonies [26]:

*People know about my work because I always spread information about malaria, diarrhoea, high temperature and respiratory infections; and I say these diseases can be cured by taking our drugs (…) I often talk about my work during religious rituals or wedding ceremonies* (V4_eVMW)*.*

The analysis of interviews with forest goers and caregivers indicates these dissemination activities and practices were effective in our study sample. The majority of participants (*n* = 41/51) were familiar with the local VMW and had a good understanding of their services. Many respondents (*n* = 17) reportedly received relevant information at village meetings, confirming these were crucial to deliver key messages. However, some issues emerged. In one expanded role village, the chief and four caregivers were not aware that the local VMW could manage child diseases in addition to malaria or provided unclear explanations. In another village, the VMW was concerned that awareness was low in his community because many residents were “newcomers” or lived in more remote locations and therefore could not be reached by communications about the programme. Finally, two participants pointed out that the signboard in front of the house where the VMW lived was a useful reminder. However, we observed that signboards were missing in two villages. One VMW explained that the rain had deleted the message and the supporting material had become rusty. In addition, one forest goer in another village said he was illiterate and could not read the text in the signboard.

### Accomodation

The capacity of providers to meet users’ needs in terms of hours of operation is another important dimension of access to medical care. In our study locations, we found differing patterns and perceptions, depending on individual and contextual variables. Four VMWs said they could meet patients any time. One VMW, for example, stressed he was “never away from home” and could see patients round the clock:

*Villagers can come and see me whenever they want to. If they come at night, I am here for them. Some patients told me they were not welcomed in other places at night. They go around and try to find a doctor somewhere else, but they often come back to me as I always welcome them* (V2_VMW)*.*

In addition, two VMWs reported they would occasionally visit patients at their homes, if someone called them and could not move due to ill health. However, when we questioned participants about accommodation issues, we elicited mixed responses. In one village, two respondents confirmed the VMW was usually at home taking care of her grandchildren, and they could meet her any time. But in two villages participants believed the local VMW was often away. As one caregiver reported:

*We are afraid we cannot find him* [the VMW]*. When we are sick, we cannot wait long […] because our child can get worse and we would be blamed for bringing the child too late to the doctor. We prefer to pay for the treatment so our children can recover quickly* (V5_CG02)*.*

Given the volunteer nature of VMWs, limited ability to accommodate patients may result from economic pressures and the need to undertake other activities, such as farming or running a business. In our sample, one VMW said she recently opened a shop in the market town, recognising this activity had reduced her free time to see patients in the village. In addition, three VMWs had other key roles in the community, such as deputy village chief or member of the commune council. In one of these villages, one forest goer said: “[The VMW] has a lot of work to do and also works with the NGO [*angkar*] (…) when the NGO come to the village, they need his help so he is often away. When I tried to see him, he was not at home” (V4_FG02). In the same village, a caregiver noted that “[the VMW] is responsible for the land survey. When students come to do the survey, he is very busy and doesn’t have time” (V4_CG02). As reported by six VMWs and confirmed by three participants, however, one assistant (usually a relative, as noted earlier) could ensure continuity of services when the principal VMW was busy on other activities.

### Accessibility

As many studies in public health evidenced, proximity to health facilities is a key determinant of health seeking behaviour and health outcomes, when travel costs and times are included [29]. In some of our research locations, authorities and participants said that recent works of road development had improved links with facilities outside the villages. One caregiver, for example, explained that travel times to the local health centre had reduced from several hours to just 10 min, after the dirt road was modernised in 2013. In the same village, the chief commented on the impact of these changes on medical practices:

*We didn’t use much modern medicine in the past because we didn’t have good roads. The new road was built in 2007. From 2000 to 2007, villagers still used Khmer traditional medicine, and delivered babies with the assistance of traditional remedies such as roots and leaves* (V6_VC)*.*

In other villages, however, the road infrastructure was still poor, as we experienced in our field trips. In these locations, many residents complained about long travel times to reach the nearest health centre and referral hospital, and some (*n* = 4) valued the proximity of VMWs to their houses. As one caregiver said, “he [the VMW] lives near here and the road is easy (…) so I can borrow someone’s bicycle and bring my child to his home in a short time” (V4_CG02). Further, four large villages in our sample were served with both VMWs and mobile malaria workers (MMWs). Unlike standard VMWs, who would normally receive patients at their houses, MMWs were encouraged to conduct active case detection by walking or cycling in and around the village. In one village, the MMW explained he would roam his community two or three times a week bringing along a bag with RDTs and ACT packs to identify potential malaria cases farther away from his house, including in areas near the forest fringes where many forest workers were staying overnight. However, he stressed that some areas were “out of control” due to the large size of the settlement:

*In a village like Phnom Thom* [fictional name]*, there are only 200-300 families. So it is easy. But this village is so large (…) some parts are very far and out of control. We can control only the near parts* (V5_MMW)*.*

These concerns were confirmed in interviews with forest goers (*n* = 4) whose households were located at considerable distance from the VMWs. One of them, for example, reported: “they [the VMWs] said the road is too difficult to reach my home and cannot use even the bicycle or the motorbike to get here” (V3_FG05). Similarly, another forest goer noted: “the village chief told us to see the village health worker [*pet phum*] if there is a problem, but it is difficult [because] his house is far from here” (V4_FG01). In another village, the chief explained that health providers located outside the village could be more accessible than malaria workers inside the village: “villagers go to the malaria workers if they live near their houses; if they live far, they go to the health centre or private providers outside the village”.

### Availability

Availability refers to the adequacy of providers in terms of supply of services and products. The ability of VMWs to diagnose and treat malaria was well recognised and valued by the majority of participants. Most forest goers reportedly suspected malaria [*kruhn chhan*] after the onset of symptoms, and some of them explicitly said this was a reason to use VMW services: as one forest goer noted, “the health worker [*pet*] in the village treats malaria (…) I felt chilly, so I suspected malaria; that’s why I went to get blood test with her” (V3_FG01). All forest goers were aware that malaria is caused by mosquito bites and the majority had correct knowledge of malaria symptoms, although a common misconception was that malaria could also be transmitted by drinking contaminated water. RDTs were well understood and seen as a reliable diagnostic tool by the majority of participants.

However, VMWs were reportedly not used as a first point of consultation, if patients or their caregivers had uncertainties over the cause of disease or other perceptions. In such cases, the need for a more comprehensive service, able to diagnose and treat a variety of conditions, was cited by all categories of interviewees as a reason to prioritise other providers. In one village, for example, the chief noted:

*If definitely [people have] malaria this [the VMW] is the right choice (…) but for some it is difficult because they [feel they] have gastrointestinal disease or other disease. That’s why people often skip this free service to go to another provider that costs money* (V2_VC).

In the same village, two forest goers reported similar views; one of them said he would visit the VMW only if he suspected malaria, but he would see a medical provider if malaria could be “mixed with typhoid fever”. In another village, one VMW added that patients wanted to recover quickly and would often prioritise standard health providers as “networking takes too long” (V3_VMW).

This theme also emerged in villages with expanded services. One authority pointed out that residents, including caregivers, prioritised clinics because they would provide a correct diagnosis when symptoms were not clearly attributable to a specific disease:

*If they suspect they have malaria, they go to the village health worker* [pet phum] *(…) but if villagers have fever, high temperature and headache (…) they often go to the private provider. The private provider can check if the patient has malaria, typhoid fever, or tonsillitis. These diseases have similar symptoms, so it is important to find someone who can find the right cause* (V5_DVC)*.*

This reasoning was shared by other caregivers (*n* = 3), who stressed the importance of having “blood test” to identify the health problem correctly. As one of them said, “[the VMW] has blood test only for malaria, so he knows only malaria” (V4_CG03). Likewise, a mother in another village said: “I am lazy to go [to the VMW] and moreover the blood-testing tool is only for malaria; it cannot find other diseases” (V5_CG03).

In some cases, such perceptions extended to the supply of drugs. A minority of participants said that private clinics in the market town had “many drugs” to treat many diseases. In addition, as documented in interviews with participants (*n* = 5) and two VMWs, a preference for intravenous fluid (vitamins and glucose), a practice widely used in Cambodia [30], emerged as an important driver of care seeking behaviour. As one malaria worker explained, “patients often ask me for serum because they are exhausted and want to feel better, but I have to tell them I can’t do that; I can only do blood test and give pills for treatment” (V2_MMW).

### Acceptability

In the literature on access to health services, the term acceptability is commonly used to describe the extent to which users are comfortable with social and cultural characteristics of providers like ethnicity, religion, status, and vice versa. In our locations, we found that some programme features could promote favourable reception of VMWs. First, as noted earlier and confirmed in interviews with all categories of respondents, VMWs were elected by villagers, after candidates had been shortlisted by the chief in consultation with the local health centre. One VMW pointed out: “They voted for me to be VMW. That makes me happy (…) I couldn’t say no because the villagers wanted me to do this” (V4_VMW). Second, all VMWs in our sample were long-term residents, who volunteered to help other community members. VMWs were proud of supporting their community, and some participants expressed admiration for their altruism and attitude. For example, an old woman who attended the interview with one caregiver said the local VMW was “honest and never cheated the villagers” (V4_CG02). In keeping with this report, a forest goer was grateful to the local VMW as “he is helping us with his service” (V1_FG05). Two other participants said they would offer the VMW a small compensation to demonstrate gratitude after recovery from illness.

Finally, we found little evidence that local concepts of disease, prevention, and treatment undermined the acceptability of VMWs. In our study locations, animistic beliefs informed understanding of health and illness in some cases, and traditional healers (*kru khmer*) were consulted occasionally by both caregivers and forest goers. However, medical care was generally sought when traditional practices and remedies were not effective. As one forest goer explained: “If we have already offered but we don’t seek for treatment we could get sick and die. Animism is one way [of healing], but the most important thing is to get medical treatment if we want to recover” (V2_FG08). Significantly, another forest goer in our sample was a traditional healer who contracted malaria while collecting herbal roots in a forest near the village. As reported, he initially self-medicated with an herbal infusion, but decided to visit the local VMW when he realised this remedy was not effective.

Perceptions about the status of VMWs, however, could affect the credibility of VMWs and utilisation. A number of forest goers (*n* = 5) and caregivers (*n* = 4) were reluctant to see VMWs as they had no professional skills or formal medical training; one caregiver noted, “if he were a real doctor, I would trust him” (V4_CG01). This perception was also reflected in beliefs about the quality of drugs and equipment. Despite evidence indicates that sub-standard malaria drugs in Cambodia were available mainly in the private sector [30, 31], five participants thought that private providers had better medicines. Interviews with other participants, however, indicate these perceptions could change, as trust and confidence were built over time. One VMW explained:

*[Some villagers] don’t trust us, so they go to private providers where they spend a lot of money, let’s say 100,000 or 200,000 riels* [25–50 USD]*. Sometimes they are still not recovered, so they come to see us. When they come, we prepare drugs properly and they are recovered so they have trust and stop to complain about us* (V6_eVMW)*.*

In line with this account, four participants reported that a positive experience with VMWs and successful recovery stimulated a change in perceptions. For example, one caregiver who was initially concerned the local VMW had no medical skills noted that:

*Some time ago my child was sick, and I went to see him* [the VMW] *to get him cured. He gave me some drugs, and my child recovered. After that time, I go to his home to get drug whenever my child is ill. I don’t spend money and my child gets better (…) now I really trust him* (V4_CG02)*.*

Similarly, one forest goer decided to see the VMW because he recovered from illness “three times with her” (V3_FG03); another forest goer said: “Now I believe in her 100% so if some villagers are sick, I will inform them to go there for blood test and they will recover for sure. 100% guarantee for malaria disease” (V3_FG02).

## Discussion

Our study aimed to gain a better understanding of the reception of malaria workers in rural Cambodia, with a focus on factors that may promote or discourage access to VMW services. The analysis of collected data indicates that the uptake of the VMW programme in user communities can be influenced by a wide and diverse range of factors. These include the nature of dissemination activities and their ability to reach different population groups; village topography and the changing road infrastructure; the involvement of VMWs in other community roles and activities; perceptions about the type of disease after the onset of symptoms; the need for more comprehensive diagnosis and service; perceptions about the credibility of VMWs as medical providers; length of VMW appointment. It must be noted that some of these variables may have complex effects on service utilisation. For example, the involvement of VMWs in other community roles may facilitate dissemination but may reduce the ability of VMWs to accommodate patients.

Despite the diversity of results, which speak to different programme components, a number of general points emerge from the combined analysis of themes and domains. First, findings across all domains indicate that, in our study locations, there was a good level of utilisation and awareness of VMWs, particularly amongst forest goers – the key target group for malaria control in Cambodia. However, some population groups may have fewer opportunities to benefit from malaria services either because they live in more remote locations or nonetheless are illiterate or new residents and therefore less integrated in the social and political life of the community, with limited access to key information sources and networks. Given the qualitative nature of this study, we cannot generalise these findings, and recognise that quantitative research is needed to measure the magnitude of potential challenges and the effects of different variables on VMW utilisation at the provincial and national level. Despite this limitation, the in-depth analysis of a small sample provides indications on key issues that should be given attention in future assessments. In particular, evaluations should be carefully designed to assess the impact of community health programmes on different categories of residents. In addition to standard demographic and socio-economic variables (such as gender, age, and wealth quintiles), surveys should include other key variables such as literacy (not only educational level), distance between the location of VMWs and users, length of stay in the village, and variables that can measure the exposure to different dissemination activities. Further, survey questions such as “Do you know village malaria workers?” or “Have you ever used village malaria workers?” are likely to elicit inaccurate answers, as VMWs where known locally by different definitions including “village doctor” (*pet phum*), “village health agent” (*phneakngear sokhapheap phum*), or “village health volunteer” (*anak smkrochet phum*). There was also confusion between VMWs and other types of health volunteers in the same villages (such as those responsible for vaccination programmes), requiring multiple probes to ascertain whether the question was clearly understood. Similarly, survey questionnaires should incorporate a strategic set of sub-questions to validate responses and enhance the reliability of results.

Second, our study highlights ways in which key changes in contexts of implementation may affect programme utilisation and suitability. Malaria transmission has reduced significantly in Cambodia, although forest malaria remains a concern, particularly in areas where artemisinin-resistance has been detected [32]. In addition, works of road modernisation have changed the geography of access to services in some areas. When the VMW programme was introduced, selected villages were remote not only for their spatial distance from health care facilities, but also due to the lack of adequate road infrastructure to reach them. Dirt roads were the only way in and out most villages. During the rainy season, dirt roads can become muddy and nearly impassable, making travel to the nearest health facility or market town extremely difficult. Given that malaria transmission occurs mostly in the rainy season [29, 31], access to medical care was particularly difficult when most needed. As we have seen, however, recent works for road development have improved travelling times and conditions. As a result, some villages are better connected to facilities that are located at reasonable distance, while other villages (or part of villages) are still remote. Thus, a systematic mapping of these changes would be crucial to identify areas where community health programmes are more or less useful (as a result of road development), where an intensification of specialised malaria workers is most needed (such as in the artemisinin-resistance containment zones), where a differentiation of services would be appropriate (as malaria is no longer a concern), and where the allocation of more VMWs is required (given the extension and topography of the village).

In general, our analysis is in agreement with former evaluations that have supported the scaling up of expanded VMWs who can diagnose and treat other conditions in addition to malaria [11, 33]. This would enhance the dependability of health workers in user communities and their ability to address changing health concerns and expectations. Yet, our study highlights a tension between users’ demand for more comprehensive community services and their perceptions about the inherent limitations of volunteers as providers of medical advice and care. As we have seen, residents were keen to use VMWs when they suspected malaria, but more reluctant to see them as a first point of consultation for any other suspected conditions, including child illnesses. In expanded villages, one explanation could be that the introduction of add-on services for children was recent, and perhaps had not received the same level of support and dissemination as malaria services. However, the analysis of results suggests an additional hypothesis. While VMWs were not seen as “real doctor”, many users were confident that RDTs could provide a reliable diagnosis. By contrast, the lack of “blood tests” for other conditions – as some participants noted – emerged as a barrier to utilisation. This suggests that the uptake of VMWs services may be higher when lay health workers are equipped with technologies that are seen as reliable and trustworthy, especially in emergency interventions when there is less time for trust building. Again, this hypothesis should be tested through systematic data collection and analysis, including further qualitative work on the reception of micro-technologies for community health care.

Opportunities and challenges we have discussed here are context-specific and require the development of local responses. However, the case of malaria workers in Cambodia reflects wider issues of access to health services, and particularly those associated with current changes in CHWs programmes worldwide. After a decline of interest in CHWs during the 1980s and the 1990s, over the past decade many countries have invested again in large-scale CHWs programmes to address critical gaps in the health system and inequitable access to resources. As Singh and Sachs [34] pointed out, recent programmes present key innovations in comparison with former interventions, in which CHWs were typically employed to perform more simple tasks such as health promotion and support to vaccination programmes. In particular, the development of simple, low-cost, mobile technologies such as RDTs for malaria has enabled an unprecedented expansion of the range of services that CHWs can efficiently provide, including *diagnosis* and *treatment* for a variety of diseases. In addition to the VMW programme in Cambodia, similar interventions have been established in Brazil, Ethiopia, Uganda, and many other countries [35]. Such programmes have the potential for greater health and economic impact; however, implementation is arguably more challenging as workers must *attract* users, unless active case detection and community monitoring are included in programme design. Thus, the problem of access is more central to the success of expanded interventions, and requires careful consideration at all stages of programme design and implementation. Despite the existence of a large body of literature on CHWs, this problem has not been explored thoroughly in past studies. Most qualitative works on the impact of CHW programmes have focused on direct CHW management issues, such as motivation, training, and incentives [36], but much less attention has been paid to their reception in user communities, the role of contextual factors [37] and whether equitable access opportunities exist [38]. In this paper, we aimed to contribute to a deeper understanding of these issues, relying on a conceptual framework that can guide systematic empirical work and hopefully stimulate similar research efforts in other contexts.

## Conclusions

The VMW programme in Cambodia has been a highly innovative intervention to provide essential health care in places where malaria was highly prevalent and access to services was problematic. Over the past few years, malaria transmission has reduced significantly in Cambodia, although concerns remain about the emergence of artemisinin-resistant malaria [32, 39]. While it is difficult to disentangle the effects of individual contributing factors (ranging from the ecological effects of deforestation to the impact of other interventions, such as mass distribution of bed-nets), past studies indicate that the VMW programme has been an effective means of increasing appropriate treatment for malaria in remote areas [6]. Our study also suggests that the VMW programme is an important source of health care in user communities, particularly for malaria control. Yet, we identified potential challenges that should be considered to improve access to VMWs, discussing relevance and implications for the scaling-up of current programmes, in Cambodia as in other countries.

Recognition of these challenges is particularly important in the present context of global health policy. Over the past decade, CHWs programmes have been expanded in many countries and are becoming an integral part of national health systems. Notably, a campaign was launched recently to train one million health workers in sub-Saharan Africa to reduce morbidity and avert mortality in mothers, newborn and children. Evidence indicates that this ambitious plan has great potential to improve health outcomes in a cost-effective way, and contribute to the achievement of the Millennium Development Goals [34]. However, continued progress in this area will require strategic allocation of scarce resources, especially given financial constraints in developing countries and drops in donor funding as a result of the current global economic crisis. In the process, the problem of access should be given priority, if we are to maximise impact, inclusiveness, and equity of future programmes.

## Additional files

Additional file 1: Interview guidelines forest goers. (DOCX 17 kb)

Additional file 2: Interview guidelines caregivers. (DOCX 17 kb)

## Abbreviations

ACT: Artemisinin-based combination therapy
ARI: Acute respiratory infection
CHW: Community health workers
CNM: National malaria center
eVMW: Expanded village malaria worker
MMW: Mobile malaria worker
ORS: Oral rehydradation salts
RDT: Rapid diagnostic test
VHSG: Village health support groups
VMW: Village malaria worker
