# Supplementary material for: Improving access to health care amongst vulnerable populations: a qualitative study of village malaria workers in Kampot, Cambodia
Source: BMC Health Serv Res. 2017 May 8;17:335. doi: 10.1186/s12913-017-2282-4 (PMC5423018; doi:10.1186/s12913-017-2282-4)
Supplement: Supplementary file 2 — Interview guidelines caregivers. (DOCX 17 kb) [file 12913_2017_2282_MOESM2_ESM.docx]

Additional file 2

**VMW qualitative study**

**Guidelines for In-Depth Interviews**

**Caregivers of children under the age of 5**

| **1. OPENING** | |
| --- | --- |
|  | **Could you tell me a little about yourself?**  **How long have you lived in this village for?**  **What is your main occupation at the moment?**  **How many children do you have? How old are they?** |
| **2. FEBRILE ILLNESS** | |
|  | **Thank you for this information. Could you please tell us something about last time one of your children under the age of 5 experienced febrile illness?**  **How long ago did this happen?**  **What did your child have?**  **How long was he or she sick for?**  **Did you have an idea what disease that could be?**  [IF YES]: **How did you know that?** |
| **3. CARE SEEKING BEHAVIOUR AND TREATMENT** | |
|  | **What did you do? Did you see someone?**  [IF NOT]:  **Why not? What did you do?**  [IF YES]:  **Why did you go there?**  **Was the provider far?**  [PROMPT]: **How far? How did you get there?**  **Did you have to pay the provider?**  [PROMPT]: **How much did it cost? Did you have the money?** [IF NOT]: **What did you do? Did you borrow money from someone?**  **What did the provider do?**  **What was your impression?**  [PROMPT]: **Did you feel comfortable? How could it be improved?**  **Did you see someone else?**  [IF YES]:  **Why did you go there? How much did you pay?**  **Did you have the money?** [IF NOT]: **What did you do? Did you borrow money from someone?**  **What did the provider do?**  **What was your impression?**  [PROMPT]: **Did you feel comfortable? How could it be improved?**  **Do you know of other health providers in the area?**  [IF YES]: **Why didn’t you go there?** |
| **4. VILLAGE MALARIA WORKERS** [IF RESPONDENT DID NOT MENTION EARLIER] | |
|  | **Do you know Village Malaria Workers?**  [PROBE TO MAKE SURE THE QUESTION IS UNDERSTOOD CORRECTLY]  [IF YES]:  **How do you know them?**  **Have you ever visited malaria workers?**  [IF YES]:  **Where did you meet them?**  **At what time?**  **What is your impression?**  *Dig into this, but do not suggest:*  *Competent? Conveniently located? Reliable? Available all time? Reputation? Attitude?*  **What did they do?**  **What can be improved?**  [IF THEY KNOW VMWs, BUT HAVE NEVER VISITED THEM]:  **Why not? Explain…** |
| **5. CLOSING** | |
|  | **Thank you so much…**  **Is there anything we haven’t discussed that you would like to say?**  **Can we ask you some additional information about yourself and your household?**  **-> CONDUCT DEMOGRAPHIC QUESTIONNAIRE** |
